# Hollow Silica Particles: A Novel Strategy for Cost Reduction

**DOI:** 10.3390/nano11061627

**Published:** 2021-06-21

**Authors:** Daron Spence, David A. Cullen, Georgios Polizos, Nitin Muralidharan, Jaswinder Sharma

**Affiliations:** 1 Electrification and Energy Infrastructure Division, Oak Ridge National Laboratory, Oak Ridge, TN 37830, USA; dspence@gatech.edu (D.S.); polyzosg@ornl.gov (G.P.); muralidharan@ornl.gov (N.M.); 2 School of Materials Science and Engineering, Georgia Institute of Technology, Atlanta, GA 30332, USA; 3 Center for Nanophase Materials Science, Oak Ridge National Laboratory, Oak Ridge, TN 37830, USA; cullena@ornl.gov; 4 Building Technologies Research and Integration Center, Oak Ridge National Laboratory, Oak Ridge, TN 37830, USA

**Keywords:** silica, thermal insulation, hollow particles, energy, carbon black

## Abstract

Thermal insulation materials are highly sought after for applications such as building envelopes, refrigerators, cryogenic fuel storage chambers, and water supply piping. However, current insulation materials either do not provide sufficient insulation or are costly. A new class of insulation materials, hollow silica particles, has attracted tremendous attention due to its potential to provide a very high degree of thermal insulation. However, current synthesis strategies provide hollow silica particles at very low yields and at high cost, thus, making the particles unsuitable for real-world applications. In the present work, a synthesis process that produces hollow silica particles at very high yields and at a lower cost is presented. The effect of an infrared heat absorber, carbon black, on the thermal conductivity of hollow silica particles is also investigated and it is inferred that a carbon black–hollow silica particle mixture can be a better insulating material than hollow silica particles alone.

## 1. Introduction

Thermal insulation materials are widely used in insulation applications, such as building envelopes, refrigerators, cryogenic storage of gases, water supply piping systems, high temperature fuel cells, thermal energy storage, heat exchangers, and combined heat and power systems [1,2,3,4,5,6]. Common thermal insulations include glass fiber, mineral wool, cellulose, extruded polystyrene (PS) foams, and polyurethane foams [7,8,9,10]. Their thermal conductivities range from 0.040 to 0.024 W/m·K [7,8,9,10]. Higher thermal conductivity of these thermal insulation materials results in the need of a thick layer of material for achieving a sufficient amount of insulation [7,8,9,10]. A thick layer of insulation material means higher usage of material and space [7,8,9,10]. Silica aerogels are emerging insulation materials with a thermal conductivity of ~0.014 to 0.020 W/m·K [11,12,13,14,15,16]. Unfortunately, aerogels are expensive, fragile, and hard to handle and lack scalability, hindering their widespread use [11,12,13,14,15,16]. Since aerogels have very low thermal conductivity, a thin layer of aerogels can provide the same amount of insulation that is obtained from a very thick layer of conventional insulation materials. Initially, pure silica aerogels were produced by using the sol-gel method [11,12,13,14,15,16]. Recently, several other types of aerogels, including carbon aerogels, graphene aerogels, carbon nanotube aerogels, and cellulose aerogels, have been produced [17,18,19,20,21].

A new type of insulation, hollow silica particles, is being explored as an alternative to aerogels. Hollow silica particles with a thermal conductivity of ≈0.02–0.03 W/m·K have been reported [22,23,24,25,26,27]. These particles can be used alone as thermal insulation, or they can be mixed with other materials to form a hybrid thermal insulation material [28,29]. Note that other insulation materials (e.g., fibers, foams) will not lower the thermal conductivity of another material when mixed with it to form a hybrid insulation, rather they can increase the thermal conductivity of the hybrid material [30,31]. Therefore, hollow silica particles are a universal thermal insulation material that can be used alone or in combination with other materials (i.e., they make other materials better thermal insulators). It is the trapped air inside the cavities that gives hollow particles their low thermal conductivity, and it is mainly because of the cavities that these particles lower the thermal conductivity of any material in which they are mixed. In addition to use as thermal insulation, hollow silica particles are valuable for use in batteries, drug delivery, thermal energy storage, and several other applications [32,33,34].

Hollow silica particles can be synthesized by several methods, such as by using polymer micelles, reverse microemulsions, inorganic (e.g., calcium carbonate, carbon, hydroxy apatite) particles, polymer (e.g., polystyrene; PS) particles, and bacteria as templates [22,23,24,25,26,27,35,36,37,38,39,40,41,42,43,44,45,46,47,48,49]. Similarly, some other unconventional methods, including etching of solid silica particles and spray drying a solution of solid silica particles, have also been explored [50,51,52,53]. The use of PS particles as templates is the most widely used approach because PS particles can be synthesized at low cost and with tunable size [22,23,24,25,26]. PS particle-based synthesis of hollow silica particles involves three steps: (1) synthesizing PS particles, (2) depositing silica shells on PS particles, and (3) removing the PS cores by burning or dissolving them to obtain hollow silica particles [22,23,24,25,26]. There are only a few companies that sell hollow silica nanoparticles. Nittestu Mining Co., Ltd., (Tokyo, Japan) provides hollow silica nanoparticles (diameter ≈100 nm, shell thickness = 5–15 nm) under the brand name ‘SiliNax’ [54].

Although hollow silica particles have been reported for more than two decades and would be useful in several applications, their use in these applications is not commercially viable because of their high synthesis costs. The high cost results from the low synthesis yield and high wastage of solvents. For example, 100 mL of ethanol or isopropanol is used to make ≤3.0 cm^3^ of hollow silica particles [22,23,24,25,26]. The main barrier to high synthesis yields is the incomplete optimization of the synthesis reaction conditions.

In the present work, a very high yield (≈25 times the reported methods) synthesis of hollow silica particles, with minimal wastage of solvents resulting in low cost, is reported. The cost of hollow silica particles was lowered by two approaches: (1) by increasing the synthesis yield for the amount of solvent used, and (2) by recycling the solvents. Additionally, the effect of carbon black (CB; an infrared radiation absorber) on the thermal conductivity of hollow silica particles is investigated. Although the effect of adding CB on the thermal conductivity of silica aerogels has been studied, it has not been reported before for hollow silica particles [55,56,57,58,59]. Therefore, it was considered worth investigating, as hollow silica particles are a different class of materials from aerogels. Hollow silica particles are a powder, whereas aerogels are large slabs. When CB is added to hollow particles, it can affect the thermal conductivity in three different ways: (1) by absorbing radiative heat, (2) by increasing or decreasing the air volume in the system, and (3) by increasing the contact resistance at the particle interfaces. Although the radiative heat absorption effect (1) of CB is well known in other materials, effects (2) and (3) have not been investigated before [55,56,57,58,59].

The first part of this paper presents optimized conditions for hollow silica particle synthesis. The second part presents a novel strategy (recycling of solvents) to lower the manufacturing cost of hollow silica particles. The third part presents the effect of CB (a radiation absorber) on the thermal conductivity of hollow silica particle powder.

## 2. Experimental

### 2.1. Chemicals

Isopropanol, tetraethyl orthosilicate (TEOS), styrene, 2,2′-Azobis(2-methylpropionamidine) dihydrochloride, and ammonium hydroxide (NH_4_OH, 28–30%) were purchased from Sigma Aldrich (Burlington, MA, USA).

### 2.2. Polystyrene Particle Synthesis

Polystyrene (PS) particles were synthesized by modifying a reported method [24]. In a typical synthesis, 100 mL water was heated at 60 °C for 10 min, followed by the addition of 5 mL of styrene to this hot water while stirring (600 rpm) using a magnetic stir bar. The reaction mixture was allowed to stir for 1 h before adding 2 mL of 160 mg/1 mL of 2,2′-Azobis(2-methylpropionamidine) dihydrochloride. Then the reaction was allowed to stir for 12 h. The synthesized particles were dried using an air blow drying oven and dried particles were further used as templates for the synthesis of hollow silica particles.

### 2.3. Hollow Particle Synthesis 

To make hollow silica particles, in a typical experiment, 5.33 g of PS particles (an average diameter 300 nm) were added into a solution having 100 mL of isopropanol, 30 mL water, and ammonium hydroxide (28–30%; to make solution pH ≈ 11). Then the reaction mixture was stirred for about 10 min. In the second step, 4.5 mL of tetraethyl orthosilicate (TEOS) in 3 aliquots of 1.5 mL each separated by 4 h was added. TEOS can be added in a single step instead of in aliquots. The core-shell particles, being heavy, settled down and were easy to remove from the supernatant by decanting or centrifugation (used by us). The synthesized polystyrene core silica shell particles were dried using an air blow drying oven at 50 °C. The dried core-shell particles were poured in a 300 mL crucible and were burned at 550 °C inside a Barnstead Thermolyne 47900 box furnace (Barnstead International, Dubuque, IA, USA) for 4 h. The burning removed the polystyrene core, and pure hollow silica particles were collected after cooling to room temperature.

### 2.4. SEM and TEM Studies

Scanning electron microscope (SEM) imaging was done using a Merlin 200 instrument (ZEISS, Oberkochen, Germany) available in the Center for Nanophase Materials (a Department of Energy user facility). SEM samples were prepared by depositing polystyrene particle or hollow silica particle solutions in ethanol on silicon wafer pieces, which were then attached to the SEM stubs using double-sided carbon tape.

Transmission electron microscope (TEM) imaging was performed on a Hitachi HF3300 TEM/STEM (Hitachi Global, Chiyoda, Tokyo, Japan). Samples were prepared by dropping hollow particle solutions on lacey carbon coated copper TEM grids.

### 2.5. Surface Area and Pore Size Measurements

N_2_ gas adsorption and desorption measurements were performed using a Quantachrome TouchWin (Quantumchrome Instruments, Boynton Beach, FL, USA) instrument to determine the surface area and pore size distribution of the hollow silica particle sample. The Brunauer–Emmett–Teller (BET) analysis was used to determine the surface area from the adsorption data, whereas the pore size distribution was calculated via the density functional theory (DFT) model using the desorption data.

### 2.6. Thermal Conductivity Measurements

Thermal conductivity measurements were performed by using a transient plane source (TPS 2500 S) instrument and sensor C5465 (Hot Disc, Goteborg, Sweden). Sample holder provided by the manufacturer was used. No weight was placed on the samples, i.e., thermal conductivity measurements were done on loose particles without pressing.

### 2.7. Solution Mixture Recycling

During the synthesis of hollow particles, only 90% of TEOS is deposited on the polystyrene particles and the remaining 10% remains in the reaction mixture. Therefore, in each iteration, only 90% of the TEOS that is used in the first iteration was added (as shown below).

- 1st iteration: 100 mL solution:5 g PS particles:4.25 mL TEOS;
- 2nd iteration: 85 mL solution:4.25 g PS particles:3.25 mL TEOS;
- 3rd iteration: 72 mL solution:3.60 g PS particles:2.75 mL TEOS.

## 3. Results and Discussion

### 3.1. Hollow Silica Particle Synthesis with High Yield

**Figure 1:**
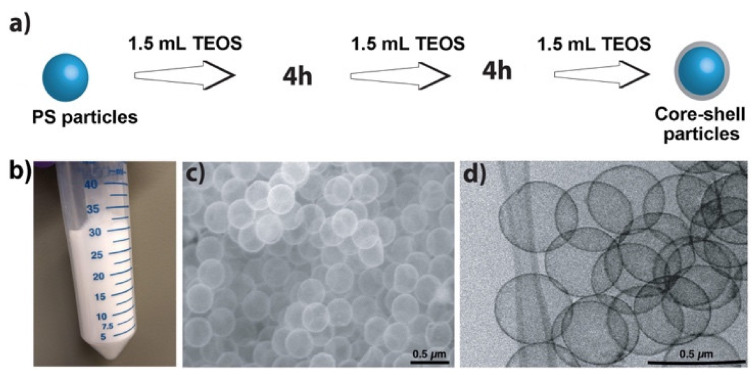
(**a**) Schematic showing the stepwise addition of TEOS at an optimum concentration to form silica shells around the PS particles at a high concentration. (**b**) Hollow particles synthesized from 100 mL of isopropanol. (**c**) SEM and (**d**) TEM images of the same hollow particles. Note: Schematic shows only the core-shell particle formation. The step of burning the PS cores to obtain hollow silica particles is not shown.

It was assumed that previous efforts to achieve high yields may have failed because very high concentrations of TEOS resulted in free silica particle formation as a side reaction. Therefore, the addition of TEOS in aliquots was chosen. Figure 1a shows a schematic demonstration of our approach of adding TEOS at specified time intervals to avoid reaching very high concentrations at any time in the reaction mixture, while adding sufficient total amounts to form shells around all the PS particles. Figure 1b shows hollow particles obtained from 100 mL of isopropanol, Figure 1c is a scanning electron microscope (SEM) image, and Figure 1d a transmission electron microscope (TEM) image of the obtained hollow silica particles.

**Figure 2:**
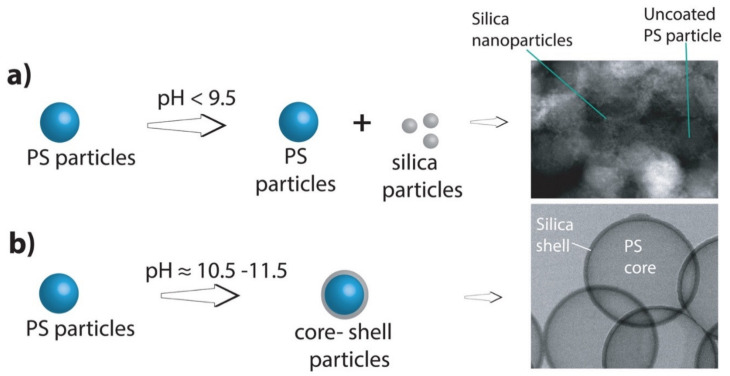
(**a**) (Left) schematic depiction of shell formation outcome when pH is below 9.5; (right) TEM image showing bare PS particles and free silica nanoparticles formed in the process. (**b**) Schematic depiction of shell formation outcome when pH is ≈11 and STEM image showing nicely formed PS core silica shell particles without any free silica particle formation. Note: Schematics show only core-shell particle formation. The step of burning the PS cores to obtain hollow silica particles is not shown.

Efforts to further increase the reaction yield by increasing the amount of PS particles (>7.5 g particles/100 mL of isopropanol) resulted in only partial shell formation on the PS particles. It was assumed that very high concentrations of PS particles increased the viscosity of the reaction mixture and, thus, hindered the uniform diffusion of TEOS. No hollow particles were formed when the isopropanol to water ratio was below 1.25. The best particles were obtained at an isopropanol/water ratio of 2.8–5.0. Note that high reaction yields can be obtained only when the reaction conditions, especially the reaction pH and TEOS amount, were fully optimized. When an insufficient amount of TEOS was added, incomplete shells were formed. Similarly, if the reaction pH was lower than 9.5, small free silica nanoparticles (≈2–10 nm) were formed. Therefore, for adequate shell formation and high yields, the pH should be higher than 9.5 (optimal pH range: 10.5–11.5) (Figure 2). TEOS was added at 4 h time intervals so that a sufficient amount of previously added TEOS can be consumed [59]. The TEOS aliquots can be added at intervals of more than 4 h but less than 10 h. It was observed that if the intervals were increased beyond 10 h, the ammonium hydroxide concentration decreased and the consumption of TEOS after subsequent aliquot additions became very slow. It was observed that the method of addition (single step or in aliquots) did not affect the final outcome of hollow silica particle formation, i.e., both methods provided the same type of particles. It was the reaction pH that controlled the shell formation. Well-formed silica shells were obtained only in a pH range of 10.5–11.5.

### 3.2. Cost Reduction of Hollow Silica Particle Manufacturing Process

To further lower the synthesis cost, for the first time, recycling of the reaction solution was demonstrated. After the shell formation, the core-shell particles settled down at the bottom of the reaction mixture container, and could be easily separated from the supernatant reaction mixture either by decantation or centrifugation (used in this work). After the core-shell particles were collected by centrifugation, the supernatant was used again to synthesize the next batch of core-shell particles by bringing the pH back to 11 (any value >10.5 gives similar results). It was observed that the pH had dropped from 11 to ≈9.0 by the end of the synthesis cycle, thus, the pH of the supernatant after a synthesis cycle was always lower than that of the initial reaction mixture. The reduction in the pH resulted from (1) evaporation and (2) consumption of ammonia as the reaction proceeded. About 85% of the solution was retrieved at each step via centrifugation, although it was noted that up to ≈95% of the solution could be retrieved. The centrifugation and supernatant use cycle was repeated three times. In each step, the amounts of PS particles and TEOS were varied to make the proportional amounts the same as in the original reaction mixture. SEM imaging showed that the quality of the particles synthesized remained the same in all iterations. Therefore, the entire process was highly useful for increasing the amount of hollow particles obtained without wasting solvent and, thus, for lowering the process cost.

**Figure 3:**
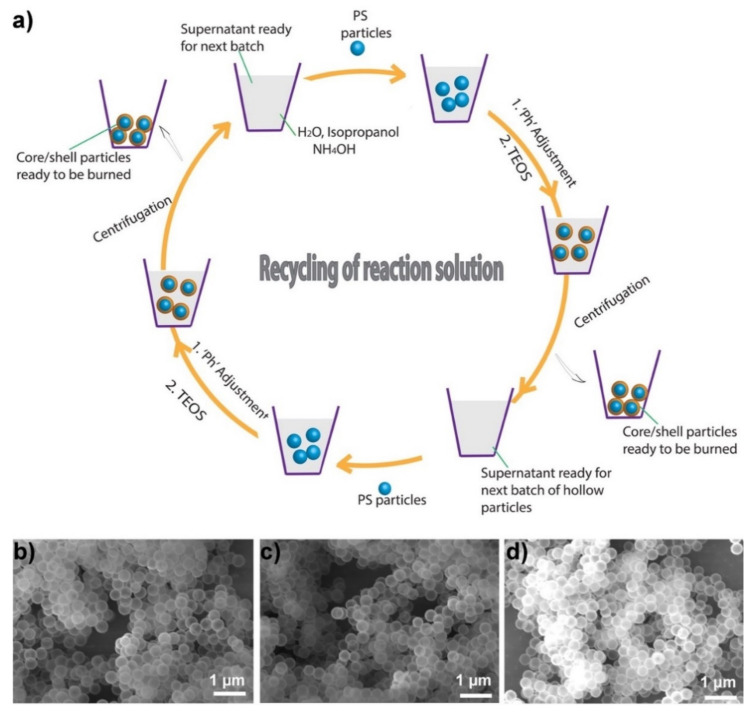
Recycling of the reaction solution. (**a**) Schematic showing the recycling steps. SEM images of the particles synthesized by (**b**) the original reaction mixture, (**c**) the first reiteration, and (**d**) the second reiteration. Note: The process was repeated only three times in this work, but the retrieved solution can be used for several more reiterations as long as the pH is maintained.

Using the same 100 mL solvent (isopropanol), approximately 75 cm^3^ of hollow silica particles were obtained, and the supernatant could still be used to synthesize the next batch of particles. Note that previously reported methods provide less than 3 cm^3^ of hollow silica particles/100 mL of solvent [22,23,24,25,26]. Therefore, our approach provided ≈25 times more hollow silica particles for the same amount of solvent if the solvent was recycled three times. However, it was calculated that, assuming 85% reaction solution recovery after each iteration (a conservative estimate, as 95% of the solution can be recovered) and extrapolating the number of iterations to six (after which the amount of reaction solution drops to about 50 mL), about 125 cm^3^ (40 times more than the previously reported methods) of particles can be obtained from the same reaction mixture (100 mL isopropanol + 30 mL water) just by adjusting the pH and adding additional TEOS. Figure 3a shows the schematic of reaction solution recycling, and Figure 3b–d are SEM images of hollow particles obtained from the original synthesis cycle, the first iteration, and the second iteration, respectively.

In a similar approach, it was observed that the reaction mixture can be used endlessly by adding additional fresh solvents (isopropanol + water) and adjusting the pH, while making the volume of the final reaction mixture equal to the original volume. The solution recycling can be repeated as long as the solution is free of any TEOS oligomers remaining from a previous batch. TEOS oligomers remaining in the retrieved solution can make the particles of the subsequent batch somewhat rough as a result of large nanograin formation. The shell formation process involves, initially, the formation of small silica nanograins (oligomers), which then attach to the PS surface to make the contiguous silica shell. To avoid the presence of any TEOS oligomers or small silica nanograins, the reaction was allowed to proceed for 12 h after the addition of the last aliquot of TEOS. The longer reaction time ensured that all TEOS was consumed [60]. Additionally, centrifugation was employed to ensure the removal of any remaining silica nanograins. High-resolution TEM/energy-dispersive X-ray spectroscopy imaging further confirmed the absence of any small nanograins in the retrieved supernatant solution used for subsequent hollow particle synthesis cycles.

### 3.3. Cost Analysis

To estimate synthesis costs, the costs of the amounts of different chemicals used in this work were compared with the extrapolated amounts of chemicals that will be used in the reported works to obtain the 1 ft^2^ × 1 cm of particles [22,23,24,25,26]. Table 1 illustrates an estimated cost analysis of hollow particle synthesis. Cost estimates show that 1 ft^2^ × 1 cm particles can be obtained at a cost of USD 1.30 by using ORNL strategy while reported methods will provide the same amount of particles at a cost of ≈USD 15. For cost analysis purposes, 130 g of polystyrene particles can be added to 2860 mL of solution (2000 mL isopropanol or ethanol + 860 mL water), followed by the addition of ammonium hydroxide and 110 mL of TEOS. After silica shell formation, ≈2450 mL of solution (≈1700 mL of isopropanol or ethanol) can be retrieved. Therefore, only 300 mL of isopropanol or ethanol will be lost by using ORNL synthesis strategy. Because the reported synthesis strategies provide a very small amount (≈3 cm^3^) of particles for 100 mL of alcohol, approximately 15 L of alcohol will be used for obtaining the same amount of particles.

By looking at the cost analysis table (Table 1), it is clear the costs of all other chemicals except the solvent are almost same in both strategies, and the cost of the solvent is the main contributing factor in the final cost of hollow particles. Our strategy reduced the amount of solvent used by recycling it and, thus, substantially lowered the manufacturing cost of hollow particles.

### 3.4. Effect of Carbon Black (CB) on the Thermal Conductivity of Hollow Particles

**Figure 4:**
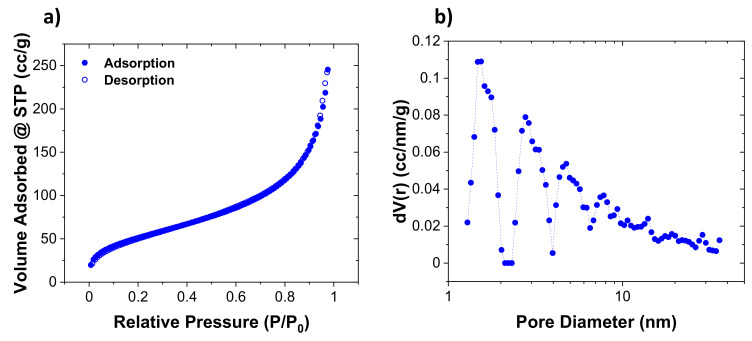
Surface area and pore size distribution measurements of a hollow silica particle sample by using N_2_ adsorption-desorption isotherms and density functional theory (DFT). (**a**) N_2_ adsorption-desorption isotherms and (**b**) the extracted pore size distributions of the SiO_2_ sample by using density functional theory (DFT).

Additionally, the effect of CB addition on the thermal conductivity of hollow silica particles was investigated. The thermal conductivity of the original particles depended upon the shell thickness, cavity size, shell quality, and whether any solid silica particle debris was present in the sample. For CB effect experiments, particles with a diameter of 300–325 nm, a shell thickness of about 6–12 nm, and internal cavity size of 285–310 nm were selected. Brunauer–Emmett–Teller (BET) analysis showed that hollow particles had a surface area of ≈115 ± 8 m^2^/g. A large portion of pores in the shell were microporous (<2 nm). Additionally, mesopores of varying sizes (large portion with 2–10 nm size and a very small portion with 2–20 nm size) were also observed. Figure 4 shows the (a) N_2_ adsorption-desorption isotherms and (b) the extracted pore size distributions of the SiO_2_ sample by using density functional theory (DFT).

**Figure 5:**
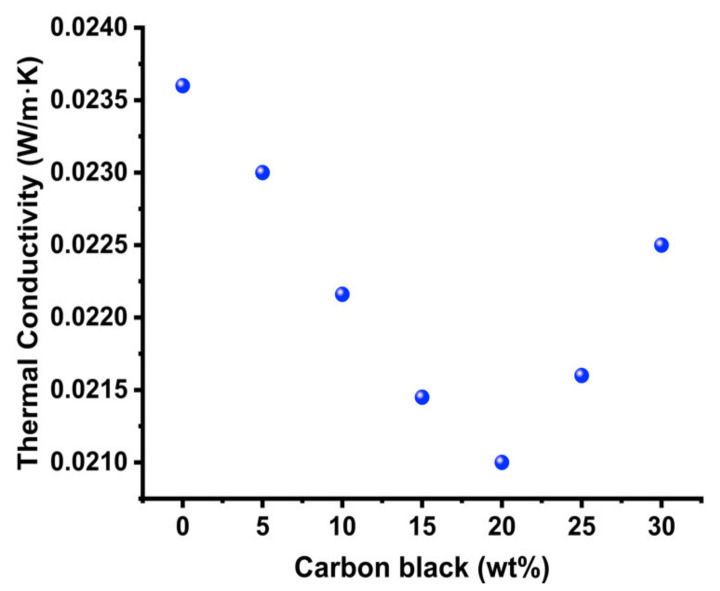
Plot showing how adding different amounts of CB affects the thermal conductivity of hollow silica particles.

The thermal conductivity of the hollow particles decreased at additions of up to approximately 20 wt% of CB and then began increasing (Figure 5). From the plot in Figure 5, it can be seen that the thermal conductivity drop is sharp in the beginning, and the decrease in thermal conductivity is more (0.0230 to 0.0221 W/m·K), from 5 wt% to 10 wt% CB addition. The thermal conductivity decreased less (0.0214 to 0.0210 W/m·K), from 15 wt% to 20 wt% CB. It appeared that in the initial stages, the addition of carbon black resulted in radiative heat absorption and, thus, showed a big decrease in thermal conductivity. In the later stages (15 wt% to 20 wt%), it seemed the sample got saturated with carbon black, and radiative heat absorption effect decreased and bottomed at 20 wt% CB addition. Beyond 20 wt%, further addition of CB may have resulted in the saturation of hollow particle sample, and no additional radiative heat absorption effect occurred. Instead of decreasing the thermal conductivity, the CB addition increased the thermal conductivity beyond 20 wt% due to pronounced effect of conductive heat transfer through carbon black particles. It must be noted, CB has been used for both decreasing the thermal conductivity (due to radiative heat absorption) and for increasing the thermal conductivity (due to increased conductive heat transfer) in different composite materials [55,56,57,58,59,61,62]. The effect of thermal conductivity can change (shape of plot, sharp or slow decline) from experiment to experiment, depending upon the degree of dispersibility, however, the trend (first decrease in thermal conductivity and then increase in thermal conductivity) remains the same.

**Figure 6:**
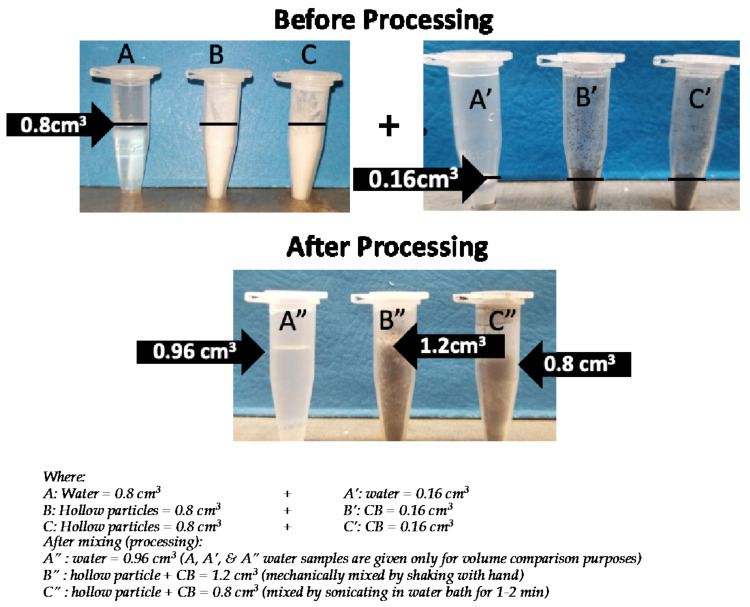
Digital photographs of different samples (hollow particles and carbon black) used in the CB effect on hollow particle thermal conductivity studies. Additionally, amounts of different samples are given in the lower part of the figure.

In aerogels, CB is known to lower the thermal conductivity by absorbing radiative energy, i.e., by mitigating the radiative heat transfer [55,56,57,58,59]. However, in powdered materials, other factors, such as a change in the air volume of the sample and increased contact resistance, can also play an important role. For example, in one experiment, a known volume (0.16 cm^3^) of CB to a known volume (0.8 cm^3^) of hollow particles. To our surprise, the combined volume (1.2 cm^3^) of CB and hollow particles was more than the sum of their individual volumes (0.96 cm^3^). Therefore, it was assumed that not only the absorption of radiative energy, but also an increase in the total air volume of the system, can contribute to lowering the thermal conductivity of a hollow silica particle and CB mixture. It is worth mentioning that hollow or porous materials have low thermal conductivity due to the air (thermal conductivity ≈ 0.024 W/m·K) inside their cavities or pores. Any increase in air volume generally results in a corresponding decrease in the thermal conductivity of a material. It appears that the increase in total volume resulted from the intercalation of CB nanoparticles between hollow particles and, thus, from the increased spacing between the hollow particles. Photographs of samples used for measuring the volumes of CB, hollow silica particles, and the combined sample are provided in Figure 6 (B, B’, & B”). Additionally, in aerogels, the lowest obtainable thermal conductivity was obtained by adding a small amount (≈10 wt%) of CB, whereas in hollow particles, the lowest thermal conductivity was achieved at quite a high amount (20 wt%) of CB (similar to the case for powdered aerogels) [55,58,59]. The difference in the results arises from the differences in the microstructures of aerogels (monolithic, consisting of 2–5 nm nanoparticles) and hollow particles (particulate, larger particle size), and from other factors. Radiative absorption plays the main role in the conductivity of aerogels, whereas increased air volume and contact resistance, along with radiative absorption, may affect the thermal properties of a CB–hollow particle mixture.

**Figure 7:**
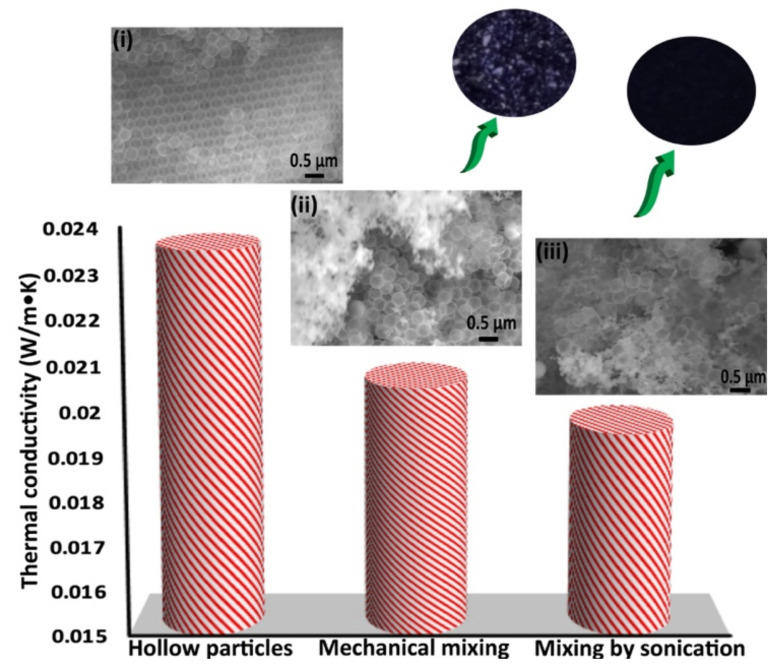
A plot showing the effect of CB dispersion on the thermal conductivity of a hollow particle–carbon black mixture. Insets (i), (ii), and (iii) are SEM images of hollow particles alone, a mechanically mixed hollow particle–CB mixture, and an ultrasonically mixed hollow particle–CB mixture, respectively. Sub-insets: Photos of mechanically and ultrasonically mixed CB and hollow particles.

To understand how CB dispersion affects the thermal conductivity of a CB–hollow particle mixture, another sample ‘C’, in which the only difference was the method of CB dispersion, was created. In sample ‘B’, the CB was dispersed mechanically by shaking the sample without adding any solvent. In sample ‘C’, ethanol was added and the sample was sonicated, followed by drying. Figure 7 bar chart shows the effect of the CB dispersion method on the thermal conductivity of hollow particle CB sample. The thermal conductivity of sample ‘C’ (CB dispersed by sonication) was 19.5 W/m·K, lower than the 20.5 W/m·K conductivity of sample ‘B’ (CB dispersed by mechanical mixing). Insets i–iii of Figure 7, respectively, correspond to SEM images of the hollow particles alone, mechanically mixed sample ‘B’, and sample ‘C’, mixed by sonication. Big chunks of CB appear in the mechanically mixed sample (inset ii), while small CB chunks are seen in the sample mixed by sonication (inset iii). Similarly, corresponding sub-inset photos clearly show that mechanical mixing did not disperse the CB completely, but sonication in ethanol resulted in better dispersion.

The improvement in thermal conductivity resulting from better dispersion of CB seems due to an increased number of interfaces between the CB and the hollow particles. It is known that an increase in the number of interfaces can increase the total contact resistance (or Kapitza resistance) of a sample [63]. This experiment indicates that more thorough CB dispersion increased the contact resistance by increasing the number of silica–CB interfaces, which helped further lower the thermal conductivity of the mixture. Since the CB amount is the same in both cases, it was assumed that radiative heat absorption is the same in both cases. Therefore, it appears that a combination of factors—increased air volume, increased contact resistance, and increased radiative absorption—lowered the thermal conductivity of the hollow particle–CB mixture. Among all these factors, it seems that the increased contact resistance had a more prominent effect than the increased air volume, because the sonicated sample ‘C’ had a smaller final volume (samples C, C’, & C” in Figure 6), but lower thermal conductivity.

## 4. Conclusions

A synthesis strategy that can provide very high yields of hollow silica particles compared with previously reported methods was demonstrated. The critical requirement for a high synthesis yield was to control the reaction pH and the amount of silica precursor. For the first time, the recycling of the reaction solution, which further lowers the synthesis cost of the hollow silica particles, was also demonstrated. Additionally, it was found that good dispersion of CB is critical to achieve the maximum reduction in the thermal conductivity of the powdered material. It is anticipated that this work will open opportunities for a number of applications requiring large amounts of inexpensive hollow silica particles. Additionally, the recycling of reaction solutions can be applied in other similar particle synthesis strategies. The findings regarding the addition of CB open new avenues to investigate the effects of opacifiers on powdered materials. They include new effects that may be related to an increase in air volume and contact resistance, which were not considered in previous studies.

## Figures and Tables

**Table 1:** Cost analysis of ORNL hollow silica particle manufacturing process compared with the currently reported processes [22,23,24,25,26].

Item	Unit Price (USD)	Required Amount ^a^		Cost (USD) ^a^	
		ORNL	Reported ^b^ [22,23,24,25,26]	ORNL	Reported
Styrene	1.5/1.1 L	200 mL	200 mL	0.3	0.3
Catalyst	5/kg	5 g	5 g	0.03	0.03
Alcohol (ethanol/isopropanol)	0.9/L	300 mL	15 L	0.27	13.5
Ammonium hydroxide	0.50/L	25 mL	1.25 L	0.0125	0.625
Tetraethyl orthosilicate	1.9/L	100 mL	100 mL	0.20	0.19
Electricity	0.10/kWh	5 kWh	5 kWh	0.50 ^d^	0.50
Total manufacturing cost ^c^				1.31	15.14

^a^ Values are based on a sample volume equivalent to 1 ft^2^ × 1 cm, ^b^ Reported amounts of chemicals ae estimated by extrapolating the amounts of different chemicals to get 1 ft^2^ × 1 cm from references [22,23,24,25,26], ^c^ Does not include labor, ^d^ Electricity consumption will be lower in ORNL strategy because of high initial yield, however we included similar costs for both manufacturing processes.
